# “THE ONLY WAY FOR US TO STAY SAFE IS TO HIDE”: THE EXPERIENCES OF OLDER CHINESE IMMIGRANTS DURING COVID-19 IN NYC

**DOI:** 10.1093/geroni/igad104.2343

**Published:** 2023-12-21

**Authors:** Yiyi Wu, Mark Brennan-Ing, Ruth Finkelstein

**Affiliations:** Hunter College, City University of New York, New York City, New York, United States; Hunter College, CUNY, New York City, New York, United States; Hunter College, City University of New York, New York City, New York, United States

## Abstract

The COVID-19 pandemic disproportionately affected older adults, who were at heightened risk for COVID-19 morbidity and mortality, as well as social isolation. Challenges to accessing health information and vaccine resources were amplified among older immigrants due to language barriers, lack of established social networks, and experiences of systemic discrimination. Considering the surge of anti-Asian violence across the country and the high-profile violent incidents targeting Asian older adults, our analysis focuses on the lived experiences of older Chinese immigrants during the pandemic in New York City (NYC). To describe the support needed during the pandemic to inform service delivery, we conducted in-depth interviews with 9 low-income Chinese adults over 65 about their experiences in coping with COVID-19. Interviews were transcribed and subjected to inductive thematic analysis. Results show that driven by the pro-vaccination and science norms in China, participants shared an overall positive attitude towards vaccines and exhibited extraordinary resilience to COVID-misinformation. However, COVID-19 and anti-Asian violence amplified each other. Fear of contracting COVID-19 due to perceived age-related vulnerability and fear of anti-Asian violence were prevalent, leading to self-protective withdrawal and deferred health care. Many expressed concerns about traveling to public places alone. Deeply frustrated with the government’s role in controlling violent crimes, some participants decided to stay home indefinitely to evade trouble. Mechanisms to draw out people who have socially withdrawn are needed. Senior service providers in NYC should initiate discussions about perceived safety and implement peer buddy systems to enhance post-pandemic quality of life for Asian older adults.

